# Molecular Characterization of *Thymus capitellatus* Extracts and Their Antioxidant, Neuroprotective and Anti-Proliferative Activities

**DOI:** 10.3390/ijms232315187

**Published:** 2022-12-02

**Authors:** Carlos Martins-Gomes, Jan Steck, Judith Keller, Mirko Bunzel, Fernando M. Nunes, Amélia M. Silva

**Affiliations:** 1 Centre for Research and Technology of Agro-Environmental and Biological Sciences (CITAB), Cell Biology and Biochemistry Lab., University of Trás-os-Montes and Alto Douro (UTAD), Quinta de Prados, 5001-801 Vila Real, Portugal; 2 Chemistry Research Centre-Vila Real (CQ-VR), Food and Wine Chemistry Lab., University of Trás-os-Montes and Alto Douro (UTAD), Quinta de Prados, 5001-801 Vila Real, Portugal; 3 Department of Food Chemistry and Phytochemistry, Institute of Applied Biosciences, Karlsruhe Institute of Technology (KIT), Adenauerring 20a, 76131 Karlsruhe, Germany; 4 Department of Chemistry, School of Life Sciences and Environment, University of Trás-os-Montes and Alto Douro (UTAD), Quinta de Prados, 5001-801 Vila Real, Portugal; 5 Department of Biology and Environment, School of Life Sciences and Environment, University of Trás-os-Montes and Alto Douro (UTAD), Quinta de Prados, 5001-801 Vila Real, Portugal

**Keywords:** *Thymus capitellatus*, phytochemical profiling, antioxidant activity, anti-proliferative activity, cytotoxicity, neuroprotection

## Abstract

*Thymus capitellatus* Hoffmanns & Link is an endemic species of the Iberian Peninsula listed as near-threatened, due to its restricted geographical distribution, occurring mainly in Portugal’s mainland. In this work, we detail for the first time *T. capitellatus* extracts’ phytochemical composition, as well as an evaluation of bioactivities to point out potential health benefits. Aqueous decoction (AD) and hydroethanolic (HE) extracts were obtained, both rich in flavonoids. However, quercetin-(?)-*O*-hexoside was identified as the main compound in *T. capitellatus* HE extract, while the phenolic acid rosmarinic acid was the main component of AD extracts. In addition, HE extract presents significant amounts of salvianolic acids and of the terpenoids oleanolic and ursolic acid. Both extracts showed antioxidant activity, evaluated by their capacity to scavenge ABTS and superoxide radicals, as well as an ability to prevent lipid peroxidation. AD extracts were also effective in scavenging hydroxyl and nitric oxide radicals. As potential functional foods, *T. capitellatus* extracts presented neuroprotective and anti-diabetic activity, in addition to time- and dose-dependent anti-proliferative activity against Caco-2 (colorectal adenocarcinoma) and HepG2 (hepatic carcinoma) cells. HE extract presented higher cytotoxicity than AD extract, and HepG2 cells were more resistant than Caco-2 cells. After 24 h exposure to HE extract, the IC_50_ values were 330 μg/mL and 447 μg/mL for Caco-2 and HepG2 cells, respectively. *T. capitellatus* has potential as a functional food or as a source of bioactive molecules. These results also highlight the need to preserve species with as yet unknown molecular compositions and potential medicinal applications.

## 1. Introduction

The concept of functional foods began in Japan in the 1980s, providing a new approach to dietary habits, in which through the consumption of specific products, in addition to the food ingested to fulfil the basic nutritional intake, an individual could improve his well-being, and where functional foods play a preventative role against various pathologies [1]. Following an increase in publications of scientific articles related to functional foods in the last few decades [1], consumers’ adherence to natural products-based items has also increased, as a growing percentage of the population has exhibited increased awareness toward the prevention of chronical diseases such as diabetes or cardiovascular and neurodegenerative pathologies. Irrefutably linked to functional foods is the use of herbal products, and among them medicinal and aromatic plants, such as species of the *Thymus* L. genus. For example, in the food industry, the antioxidant and antimicrobial activity of these plants increases the storage time of many food products [2], being incorporated in dairy, meat or fish products, thus avoiding the use of synthetic additives [2,3,4]. In the pharmaceutical industry, *Thymus zygis* L. essential oils and/or extracts are used in the formulation of expectorant and anti-cough syrups [3].

However, to address the increasing demand from several industries for new natural-based products, nutraceuticals and functional foods, a growing number of plant species must be screened for their potential bioactivities. Within the *Thymus* genus, some species are commonly consumed, e.g., *Thymus vulgaris* L., *Thymus mastichina* L. or *Thymus* × *citriodorus* (Pers.) Schreb., and so arise as a focus for various research topics due to their market value. However, a window of opportunity has been created for the study of uncharacterized species (both for their phytochemical composition and bioactivities), whose potential applications are yet to be unveiled. In the specific case of Portugal, there is an opportunity to draw attention to the preservation and valorization of the country’s rich flora, as several endemic thyme species are listed as near-threatened or as vulnerable species due to anthropogenic action and climate change. *Thymus carnosus* Boiss., *Thymus capitellatus* Hoffmanns & Link, or *Thymus albicans* Hoffmanns & Link are some of the species that due to their restricted distribution, limited to the Iberian Peninsula, are listed in International Union for Conservation of Nature (IUCN) Red List of Threatened Species [5].

Roxo et al. (2020) [6] provided a scientific background to the traditional use of *T. albicans* to treat inflammation as well as its potential use as an antifungal agent. The essential oil of *T. albicans* exerts anti-inflammatory activity at non-cytotoxic concentrations by inhibiting the activity of inducible nitric oxide synthase (iNOS), and consequently reducing nitric oxide (NO) levels, as well as antifungal activity against various *Candida*, *Microsporum*, *Trichophyton* or *Aspergillus* strains, highlighting its potential applications in pharmaceutical industry. Another near-threatened *Thymus* species, *T. carnosus,* whose phytochemical composition of its extracts was recently published, was revealed to be a promising source of bioactive compounds such as salvianolic, ursolic and oleanolic acids [7]. Besides their anti-inflammatory potential [7], *T. carnosus* extracts exhibited anti-proliferative activity against several human cancer cell lines, with a particular emphasis on the Caco-2 cell model, where the mechanism of action was linked to apoptosis and cell cycle arrest, thus proving a potential application for these species [8].

Ethnobotanical surveys on *Thymus capitellatus* report the traditional use of this species for their aromatic properties, for medicinal, cosmetic and culinary purposes [9]. Both their flowers and leaves have been used to scent perfumes and soaps or have been boiled with sugar/honey to produce an anti-cough syrup [9].

As for many species of medicinal and aromatic plant, essential oils obtained from *T. capitellatus* have been the main focus of phytochemical analysis and bioactivity studies. Plant material collected in the Estremadura and Ribatejo regions (Portugal) in both vegetative and flowering stages presented the same chemotype, with essential oils rich in 1,8-cineole, borneol, camphene, α-pinene and sabinene [10,11,12]. Regarding the bioactivities, an anti-leishmania effect [10], tested at concentrations not cytotoxic to mammalian cell cultures, and anti-fungal [11] activity were found. Concerning *T. capitellatus* extracts, only a few studies can be found in the literature, providing an incomplete characterization of *T. capitellatus* extracts. Three studies, dating from 1981 to 1988, report the presence of vicenin-2, luteolin, apigenin, eriodictyol, naringenin, caffeic acid and rosmarinic acid in methanolic extracts, phenolic compounds commonly described in most thyme species [13,14,15]. Tavares et al. [16] identified a luteolin glycoside derivative as a major compound in *T. capitellatus* hydroethanolic extract. Neuroprotective potential can be highlighted as being common to both essential oils and extracts, as both inhibited acetylcholinesterase activity with IC_50′_s ranging from 12 to 561 µg/mL for essential oils, depending on the harvest location, and 490 µg/mL for the hydroethanolic extract [16].

Therefore, in this study we aimed to fully characterize *T. capitellatus* aqueous and hydroethanolic extracts concerning their molecular phytochemical composition, and to further screen for relevant bioactivities. Antioxidant, anti-proliferative, neuroprotective, anti-aging, and anti-diabetic activities were evaluated to provide a scientific background for its use as a functional food, within a sustainable cropping model, preventing the loss of both valuable information and biodiversity.

## 2. Results and Discussion

In the present study, we aimed to provide a complete phytochemical characterization of *T. capitellatus* aqueous decoction (AD) and hydroethanolic (HE) extracts, obtained by exhaustive extraction, for the first time. Despite being part of the *Thymus* genus, in which there are several species that are already well-described from the phytochemical point of view and used in the human diet, the phytochemical composition, antioxidant activity and bioactivities of *T. capitellatus* extracts are only partially studied, with a reduced number of publications reporting the potential of this species. Contributing to this factor is the fact that *T. capitellatus* has a near-threatened status and has a restricted geographic distribution; however, a detailed description of the phytochemical composition of its extracts as well as its potential bioactivities may increase the interest in the preservation of this plant species as well as its sustainable use and cultivation.

### 2.1. Extraction Yield, Total Phenolic, Total Flavonoid and Ortho-Diphenols Content

Regarding the extraction yield (Table 1), AD and HE extracts yielded 15.82 ± 2.30% and 16.84 ± 2.43% (% *w*/*w*), respectively. Compared with other *Thymus* species extracted with the same method, the AD yield value is similar to that of AD extracts of *T. pulegioides* (14.55% [17]), and is higher than the yields of *T. mastichina* (9.32% [18]) and *T.* × *citriodorus* (9.35% [19]) AD extracts, but is lower than those reported for *T. carnosus* (21.2 % [7]), *T. vulgaris* (25.65% [20]) or *T. zygis* (29.70% [3]) extracts. For *T. capitellatus* HE extracts, the yield is in line with the values obtained for *T. mastichina* (13.78% [18]) and *T.* × *citriodorus* (14.05% [19]), but lower than those obtained for the remaining species listed above. Comparing both extraction methods, HE extract presented a higher yield mean value, but this was not statistically different (*p* > 0.05). The edaphoclimatic characteristics of the harvest location, the plant’s vegetative phase, as well as the harvest date may be the main factors contributing to these variations within plants of the same genus. *T. capitellatus* aerial parts were collected in November, and the other species listed above were harvested between April and October. Additionally, *T. capitellatus’* habitat is located at a much lower altitude than the harvest location of the other species used for comparison, with smaller seasonal temperature variations and distinct edaphoclimatic conditions.

Concerning total phenolic content (TPC), *T. capitellatus* extracts’ TPC is not dependent on the extraction method, as the TPC values are identical, i.e., 23.77 ± 0.84 and 23.72 ± 0.91 mg CAE/g DP for AD and HE extracts, respectively (Table 1). Frequently, the exhaustive hydroethanolic extraction allows a higher retrieval of phenolic compounds from the plant material when compared to AD extracts, due to sequential extraction steps and higher solubility in ethanol [7].

However, Folin–Ciocalteu reagent, beyond reacting with phenols, also reacts with other water-soluble constituents such as sugars or proteins [21], which could explain the similar TPC values for both AD and HE extracts (Table 1). Within the *Thymus* genus, it was observed that the extraction method affected TPC, for example *T. pulegioides* (AD: 26.12 ± 0.91 mg CAE/g D.P.; HE: 56.11 ± 5.6 mg CAE/g D.P. [17]) or *T. mastichina* (AD: 12.51 ± 2.97 mg CAE/g D.P.; HE: 24.61 ± 0.67 mg CAE/g D.P. [18]), extracted and evaluated for TPC according to the same methodologies; however, *T. fragrantissimus* showed identical TPC for AD and HE extracts (26.09 ± 1.63 and 29.09 ± 3.05 (mg CAE/g D.P.; *p* > 0.05) [22]). AD extracts of *T. capitellatus* exhibited higher TPC values (23.77 ± 0.84 mg CAE/g DP) than those quantified in AD extracts of *T. mastichina* (12.51 ± 2.97 mg CAE/g DP [18]) or *T.* × *citriodorus* (15.53 ± 4.75 mg CAE/g DP [19]), and values in line with those obtained for *T. vulgaris* (21.56 ± 1.64 mg CAE/g DP [19]).

Concerning HE extracts, *T. zygis* 44.70 ± 1.61 mg CAE/g DP [3]) HE extract presented higher TPC values (~1.88-fold) when compared to *T. capitellatus* HE extract (23.72 ± 0.91 mg CAE/g DP; Table 1). *T. mastichina* (24.61 ± 0.67 mg CAE/g DP [18]) and *T. vulgaris* (25.12 ± 1.48 mg CAE/g DP [19]) HE extracts presented TPC values similar to that in the present work. When compared in gallic acid equivalents/g DP, the value obtained in this work for HE (65.68 GAE/g DP) extract is significantly higher than that reported by Tavares et al. (2012) [16] for *T. capitellatus* hydroethanolic (1:1, ethanol:water extracts; 12.82 GAE/DP). This difference can result from distinct extraction methods, as in this work 0.5 g of plant material was extracted using 80% ethanol (150 mL) in HE extract, with a plant:solvent ratio of 3.33 mg DP /mL, whilst Tavares et al. (2012) used 1 g of plant material and 6 mL of solvent, for a ratio of 166.66 mg DP/mL. Therefore, the lower plant material:solvent ratio improved phenolic compound extraction, resulting in a higher TPC value, which was also observed for total flavonoid content (TFC). *T. capitellatus* HE extracts obtained by Tavares et al. (2012) [16] presented a TFC value of 6.09 ± 0.28 mg catechin equivalents/g DP, a value ~4.22-fold and ~5.22-fold lower than those here reported for AD (27.56 ± 1.83 mg CE/g DP) and HE (31.81 ± 1.31 mg CE/g DP) extracts of *T. capitellatus* (Table 1), respectively. In other *Thymus* plant extracts, the HE method presented the highest extractability of flavonoids. Both AD and HE extracts of *T. mastichina* (AD: 17.37 ± 1.14 mg CE/g DP; HE: 25.44 ± 1.57 mg CE/g DP [18]) presented lower TFC when compared to *T. capitellatus* extracts, while AD extracts of *T. carnosus* (28.03 ± 2.04 mg CE/g DP [7]) and *T.* × *citriodorus* (26.51 ± 5.41 mg CE/g DP [19]) presented similar TFC values when compared to *T. capitellatus* AD extracts (Table 1). Regarding HE extracts, the values here presented are significantly lower that the values reported for *T. pulegioides* (61.75 ± 12.58 mg CE/g DP [17]) and *T. zygis* (61.52 ± 2.37 mg CE/g DP [3]).

Concerning *T. capitellatus* extracts’ ODC, an extraction method-dependent pattern is observed, where HE extracts present a higher ODC value, according to those obtained for *T. vulgaris* (AD: 16.13 ± 1.48 mg CAE/g DP; HE: 23.41 ± 0.56 mg CAE/g DP [19]) or *T.* × *citriodorus* (AD: 16.26 ± 1.92 mg CAE/g DP; HE: 22.97 ± 3.30 mg CAE/g DP [19]) HE extracts. The extraction method-dependent effect is mainly explained by the higher extractability of phenolic acids, such as rosmarinic acid, by alcohols, and thus reflecting ODC. The results for *T. vulgaris* [19] and *T.* × *citriodorus* [19] AD extracts’ ODC is lower than the value here presented for *T. capitellatus* (21.36 ± 0.80 mg CAE/g DP; Table 1), but are similar to the value presented for *T. capitellatus* HE extract (24.60 ± 0.61 mg CAE/g DP; Table 1).

### 2.2. Profiling and Quantification of Individual Compounds by HPLC-DAD and HPLC-ESI-MS^n^

**Figure 1:**
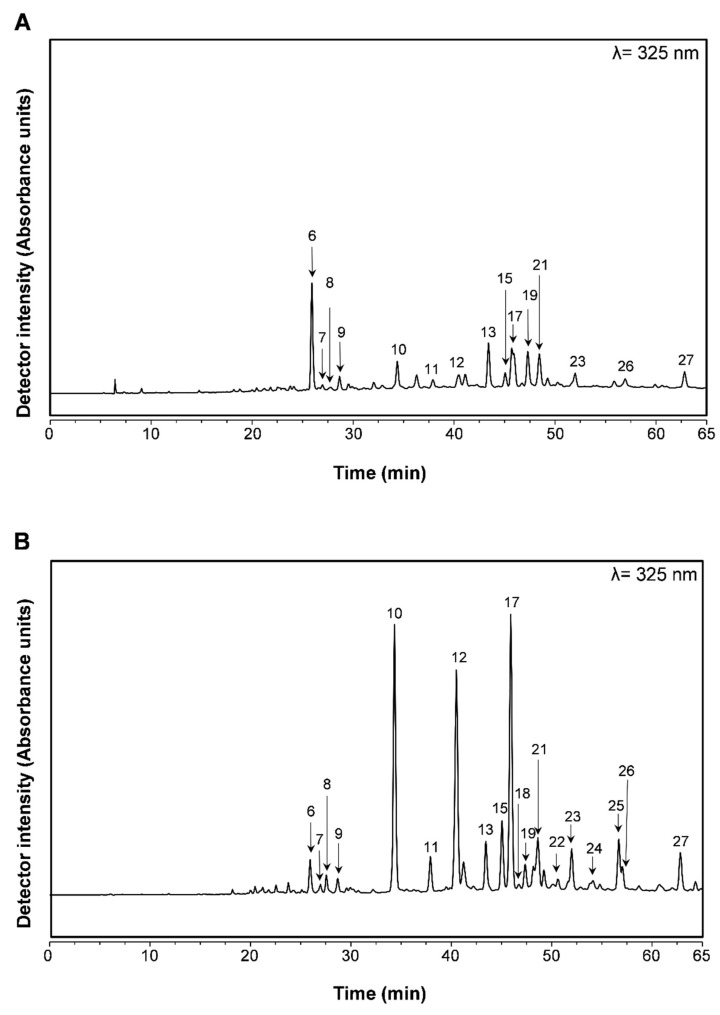
Phenolic profile of *T. capitellatus* aqueous decoction (**A**) and hydroethanolic (**B**) extracts obtained by HPLC-DAD analysis. For peak identification, please refer to Table 2.

*T. capitellatus* extracts’ phytochemical composition has been briefly approached through qualitative analysis, reporting a composition rich in flavonoids and phenolic acids [13,14,15,16], and therefore similar to other extracts obtained from other *Thymus* species. However, this information is insufficient to establish the species as a potential functional food and source of nutraceuticals. Therefore, in this work, a full qualitative and quantitative analysis of *T. capitellatus* aqueous and hydroethanolic extracts is described (Figure 1 and Table 2) for the first time.

As highlighted in *T. capitellatus*’ phytochemical composition, a high content of flavonoids is observed (Table 2), representing the majority of phenolic compounds identified and quantified by HPLC-DAD-ESI-MS^n^, representing 66% of AD extract and 76% of HE extract. When compared with extracts obtained using the same methodologies, only *T. pulegioides* [17] and *T. zygis* [3] present similar values for AD extracts (61% and 58%, respectively), while species such as *T. carnosus* (9.6%) [7], *T. mastichina* (35.4%) [18] or *T. vulgaris* (33.20%) [19] present significantly lower flavonoid contents. For all of these species, HE extracts present a significantly lower content in flavonoids when compared to *T. capitellatus* HE extracts. As a contributing factor, we can note the reduced content of rosmarinic acid and salvianolic acids in *T. capitellatus* extracts. While *T. capitellatus* AD and HE extracts presented 6.37 and 21.71 mg rosmarinic acid/g extract, respectively (Table 2), the content of rosmarinic acid in *T. mastichina* [18] or *T. vulgaris* [19], *T.* × *citriodorus* [19], *T. pulegioides* [17] and *T. zygis* [3] ranged between 14.07 and 58.5 mg/g of AD extract and 48.7 and 151.9 mg/g of HE extract. Only *T. carnosus* presented a content of rosmarinic acid (AD: 4.4 mg/g and HE: 29.1 mg/g) similar to that of *T. capitellatus* (Table 2), but the content of total phenolic acids still represents the majority of the total phenolic compounds due to a high content of salvianolic acids A isomer and K, with values 35-fold higher for salvianolic acid A isomer (Compound **13**; Table 2) and 41-fold for salvianolic acid K (Compound **21**; Table 2) when compared to HE extracts of both species. Additionally present in *T. capitellatus* extracts, a second salvianolic acid isomer was identified and listed as K isomer (Compound **24**; Table 2), which was also observed in *T. mastichina* extracts [18], but not quantified. An extraction method-dependent effect was observed for rosmarinic acid, salvianolic acid A isomer and salvianolic acid K isomer, and therefore also in the total phenolic acids, correlating with the results obtained for ODC content (Table 1).

Caffeic acid (Compound **6**; Table 2), commonly observed within the *Thymus* genus, was also identified and quantified in both aqueous and hydroethanolic extracts. Although presenting higher extractability with alcoholic solvents, the content of caffeic acid is higher in AD than in HE extract, which was also reported for *T. zygis* extracts. This could be explained by the use of high temperatures during AD extraction, which results in the partial hydrolysis of rosmarinic acid and thus in the increase in caffeic acid content [3].

Concerning flavonoids, *T. capitellatus* extracts stand out as a source of glycosidic derivatives of common flavonoids (Table 2). Luteolin, for example, although being present as aglycone (Compound **27**; Table 2), can also be found in the form of five glycosidic derivatives in both AD and HE extracts, while four quercetin derivatives and two derivatives of apigenin, eriodictyol and chrysoeriol were also detected (Table 2).

Apigenin is present in a di-glucoside derivative, where the sugar moiety is linked to apigenin through a *C*-link, commonly known as vicenin-2 (Compound **3**; Table 2), and also through a *O*-link to a single hexoside (Compound **18**; Table 2), both being compounds commonly found in the *Thymus* genus, as for example in *T. mastichina* [18]. Two isomers of eriodictyol-(?)-*O*-hexoside were found in *T. capitellatus* AD and HE extracts, with Compound **9** being the most abundant (Table 2), with 4.99 ± 0.61 mg/g HE extract and 2.43 ± 0.79 mg/g AD extract. Eriodictyol-(?)-*O*-hexoside was also identified in *T. zygis* [3] extracts, but only one isomer was identified, contrarily to *T. capitellatus* extracts, which contain two different derivatives where the hexoside moiety is linked in different positions within the aglycone. *T. zygis* AD extract presented 4.80 ± 0.17 mg/g extract, and the HE extract displayed 8.77 ± 1.13 mg/g extract [3], both higher than the values here presented for *T. capitellatus* AD and HE extract, respectively. The presence of both isomers was also reported by Pereira et al. (2013) in *T.* × *citriodorus* [23] and by Ziani et al. (2018) in *T. algeriensis* [24]. Of particular interest, chrysoeriol-(?)-*O*-hexoside-hexoside was identified in *T. capitellatus* extracts (Table 2), through the loss of fragments with 162 Da and 323 Da from the pseudo-molecular ion with *m*/*z* equal to 623, which represent the loss of one or two sugar residues, respectively, thus clarifying the structure as a disaccharide linked to the aglycone instead of two monosaccharides linked in different positions.

Regarding quercetin derivatives, two derivatives deserve special attention, as their occurrence in thyme species is rarer when compared to quercetin linked to an hexoside or hexuronide moiety. Compound **15** (Table 2) presents a pseudo-molecular ion with an *m*/*z* of 625 and ion fragments with *m*/*z* 463, which correspond to the loss of the monosaccharide moiety (loss of 162 Da) from the main ion (*m*/*z* 625), and the fragment *m*/*z* 301 and 323, corresponding to the aglycone (*m*/*z* 301) and the loss of the (caffeoyl)-hexoside moiety, which comprises the monosaccharide (162 Da) and caffeoyl (caffeic acid with the loss of an H_2_O molecule (162 Da). This fragmentation pattern and the same compound were identified in *Equisetum giganteum* [25], which also served as a literature comparison. Compound **22** (Table 2) presents the same fragmentation pattern as rutin (quercetin-(?)-*O*-rutinoside), but a comparison of UV/Vis spectra with a rutin (quercetin-3-*O*-rutinoside) commercial standard excluded this possibility. Therefore, due to the loss of fragments with 146 Da and 162 Da, corresponding to the loss of deoxyhexoside and hexoside moieties, respectively, either the rutinose is linked in a different position (differing from quercetin-3-*O*-rutinoside), or a different disaccharide and not rutinose is linked to the aglycone. Among luteolin derivatives, common substitutions were found within *T. capitellatus* extracts, mainly hexoside or hexuronide fragments, as seen in Table 2. The fragmentation pattern of compound **25**, luteolin-(?)-*O*-hexoside-hexoside, allowed us to partially highlight the derivative structure, as the loss of the fragment with *m*/*z* = 323 indicated the loss of both monosaccharides simultaneously, therefore differentiating it from luteolin-(?)-*O*-diglucoside.

Quercetin-(?)-*O*-hexoside (Compound **10**; Table 2) and luteolin-(?)-*O*-hexoside (Compound **12**; Table 2) were the most abundant phenolic compounds in the HE extract. Compared to *T. zygis* [3], which is also rich in these flavonoids, *T. capitellatus* presented 7.80 times more quercetin-(?)-*O*-hexoside and 1.20 times more luteolin-(?)-*O*-hexoside.

To further unveil *T. capitellatus* HE extract’s phytochemical composition, a pentacyclic triterpenoid-targeted HPLC-DAD search was performed. From this analysis, we could confirm the presence of both oleanolic (35.77 ± 8.04 mg/g extract) and ursolic (28.97 ± 6.38 mg/g extract) acids. These terpenoids have been reported in *T. carnosus* [7], *T. pulegioides* [17] and also in *T. zygis* [3]. *T. capitellatus’* content of these terpenoids is significantly higher than that of *T. pulegioides* and *T. zygis* (0.99–3.85 mg/g HE extract). However, *T. carnosus’* content of ursolic acid was 2.59 times higher than *T. capitellatus’* content, while the content of oleanolic acid was identical in both species.

### 2.3. In Vitro Antioxidant Activity Assessment

**Figure 2:**
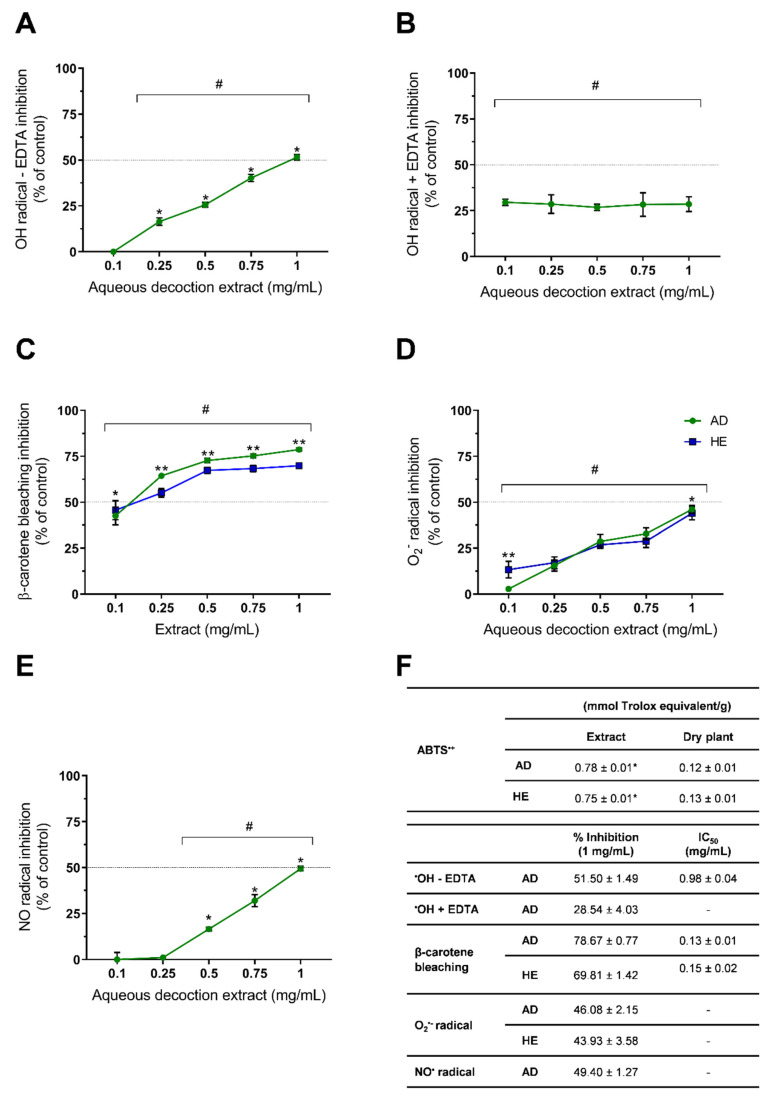
In vitro evaluation of *T. capitellatus* antioxidant activity. Hydroxyl radical scavenging (**A**,**B**), β-carotene bleaching (**C**), superoxide radical scavenging (**D**), nitric oxide radical scavenging (**E**) and respective inhibitions at 1 mg/mL and IC_50_ (**F**). Results are presented as % of radical inhibition (% of the control). Significant statistical differences are marked as (#) when compared to the control, (*) when compared with the previous and following concentration, and (**) between extracts at the same concentration, when *p* < 0.05, demonstrated by using Tukey’s post hoc test. Results are presented as mean ± standard deviation (*n* = 3).

Among the various bioactivities described for medicinal and aromatic plants, antioxidant activity is presented in the majority of studies regarding in vitro bioactivities. The *Thymus* genus presents a high number of species for which the antioxidant activity was already described, and even *T. capitellatus* antioxidant activity was briefly studied by Tavares et al. (2012), who reported the antioxidant potential of *T. capitellatus* hydroethanolic extracts against peroxyl (ROO^•^) radical (0.449 mmol trolox equivalents/g DP) and hydroxyl radical (71.2% inhibition) [16]. However, in the present research, we further investigated *T. capitellatus’* in vitro scavenging activity against other radicals (ABTS^+•^, HO^•^ (hydroxyl), NO^•^ (nitric oxide), superoxide (O_2_^−•^)) and in a β-carotene bleaching assay, whose results are presented in Figure 2.

The ABTS^+•^ scavenging by *T. capitellatus* AD and HE extracts was 0.12 ± 0.01 and 0.13 ± 0.01 mmol Trolox equivalent/g DP (*p* > 0.05), respectively (Figure 2), values similar to those obtained by the same methodology for *T. pulegioides* AD extract (0.15 ± 0.01 mmol Trolox eq./g D.P.) [17] and higher than that obtained for *T. mastichina* AD extract (0.08 ± 0.01 mmol Trolox eq./g D.P.) [18]), but lower than those of HE extracts of the same species (*T. pulegioides*: (0.34 ± 0.10 mmol Trolox eq./g D.P. [17]; *T. mastichina*: 0.20 ± 0.00 mmol Trolox eq./g D.P. [18]). As the content in total phenolic compounds is correlated with the scavenging potential of the extracts, *T. pulegioides* [17], *T.* × *citriodorus* [19] and *T. mastichina* [18] present higher TPC in HE extracts than in AD, corroborating the higher ABTS^+•^ scavenging, unlike in *T. capitellatus*, where AD and HE extracts present similar TPC values (Table 1). Although this assay is widely used to assess the antioxidant potential of various natural products, ABTS^+•^ is a chemically synthesized radical with no biological relevance, and hence its data should be used for inter-species comparison, but they provide no significant insight into the potential effects in a biological context.

Reactive oxygen (ROS) and nitrogen (RNS) species may interact with lipids, proteins, DNA and other cellular constituents, causing oxidative stress that can result in extensive cellular damage, leading to faster tissue aging, neurodegenerative pathologies and carcinogenic events [26,27]. Among ROS and RNS, ^•^OH, O_2_^−•^, ROO^•^ and NO^•^ radicals play a major role in oxidative events [27], and their direct scavenging by phytochemicals can be evaluated by in vitro methods.

Concerning hydroxyl radical scavenging by AD extracts, we obtained an IC_50_ of 0.98 ± 0.04 mg/mL in the EDTA-free (Figure 2A,F) assay, and at the highest tested concentration (1 mg/mL), inhibitions of 51.50 ± 1.49% for EDTA-free (Figure 2A,F) and 28.54 ± 4.03% for EDTA-dependent (Figure 2B,F) assays were observed. Martins-Gomes et al. (2018) [7] reported that *T. carnosus* AD extracts presented higher inhibitions at lower concentrations in the presence of EDTA, an effect reverted in the EDTA-free assay, where a dose-dependent antioxidant activity is observed. It was suggested that, due to the iron-chelating effect of EDTA, and therefore the lower radical production, a pro-oxidant effect is observed [7]. In *T. capitellatus* AD extracts (Figure 2B), the inhibition was not dependent on extract concentration, and therefore a balance between pro-oxidant and antioxidant events is reached as the concentration increases, stabilizing the maximum inhibition achieved. Without the EDTA chelating activity (Figure 2A), increasing extract concentrations interact with the hydroxyl radical that is being produced by the Fenton reaction between FeCl_2_ and H_2_O_2_. Concerning other thyme species, at 1 mg/mL of AD extracts in EDTA-free assay, *T. mastichina* (28.23 ± 3.88%) [18] and *T.* × *citriodorus* (30.59 ± 2.08%) [19] showed lower scavenging capacity when compared to the *T. capitellatus* AD extract.

As for the hydroxyl radical scavenging assay, the nitric oxide and superoxide radical scavenging activity by *T. capitellatus* extracts (Figure 2C,E, respectively) were also assessed at various concentrations (range 0.1–1 mg/mL) to further understand their potential application as an antioxidant dietary agent. For the nitric oxide assay, for concentrations higher than 0.25 mg/mL of AD extract, a dose-dependent inhibition was observed, achieving a maximum of 49.4% inhibition at 1 mg/mL (Figure 2E). This scavenging activity is higher than that obtained for AD extracts of *T. zygis* (29.32 ± 1.67%) [3], *T. carnosus* (41.79 ± 2.64%) [7] or *T.* × *citriodorus* (41.15 ± 3.64%) [19], but lower than that of *T. vulgaris* [19] AD extracts (57.61 ± 2.76%), suggesting a composition-dependent inhibition of the various radicals. Superoxide radical scavenging was performed for both AD and HE extracts (Figure 2D), and only at the lowest tested concentration (0.1 mg/mL) were significant differences observed between extracts. As seen in Table 1, TPC analysis did not present significant differences between AD and HE extracts, most likely due to the presence of other water-soluble components that reduce Folin–Ciocalteu reagent [21]. Polysaccharides, for example, may be among these compounds that present higher solubility in water when compared with ethanol, and have been demonstrated to scavenge superoxide radicals [28]. We hypothesize that at the lowest concentration tested (0.1 mg/mL), the scavenging activity is mainly due to phenolic compounds, which are present at higher concentrations in HE extract, thus presenting higher scavenging activity when compared to AD extract. As the concentrations of the extracts increase, other water-soluble compounds present in AD extracts (e.g., polysaccharides) reach a concentration which exerts antioxidant activity. Therefore, in concentrations ≥ 0.25 mg/mL, the sum of the scavenging potential of non-polyphenols plus polyphenols present in AD extract is identical to the scavenging potential of the higher concentration of polyphenols in HE extract.

A dose-dependent inhibition was observed for both extracts (Figure 2D). Superoxide radical scavenging activity reported for other thyme species presented a wide range of results, such as ~45% inhibition by 0.5 mg/mL of *T. vulgaris* [29] water extract and 90% and 77% inhibition by 0.1 mg/mL of aqueous and ethanolic extracts of *Thymus praecox* [30], respectively, both showing higher scavenging activity than *T. capitellatus* extracts at the same concentrations (Figure 2D).

Concerning the β-carotene bleaching assay, an in vitro model to assess lipid peroxidation inhibition, both AD and HE extracts of *T. capitellatus* greatly inhibited the bleaching effect, with 78.67 ± 0.77% and 69.81 ± 1.42% inhibition for AD and HE extracts at 1 mg/mL, respectively. As observed for radical scavenging assays, a dose-dependent inhibition was observed, it being relevant that at concentrations equal to or higher than 0.25 mg/mL, AD extracts perform better than HE extract in preventing β-carotene bleaching. The IC_50_ values obtained were slightly higher than the first concentration tested, being 0.13 ± 0.01 and 0.15 ± 0.02 mg/mL of AD and HE extracts, respectively. When comparing with other thyme extracts, *T. nummularius* methanolic extract presented β-carotene bleaching with IC_50_ = 6.54 µg/mL [31], a value significantly lower than that obtained for *T. capitellatus* (Figure 2C), and the authors highlighted the effect of rosmarinic acid, present in high quantities in *T. nummularius* methanolic extract and in most *Thymus* species, as this phenolic acid also presented inhibition toward the β-carotene bleaching assay [31]. Similar findings were reported by Afonso et al. (2017) for *T. caespititus* and *T. pseudolanuginosus* aqueous extracts, with 50% bleaching inhibition at 6.10 and 2.40 µg/mL [32], respectively. On the other hand, *T. mastichina* methanolic extract presented a higher IC_50_ value (0.9 mg/mL) [33]. This variation of results within species of the same genus may arise from variations in the extraction method but also from different chemical composition, which is dependent on the edaphoclimatic conditions in which the various samples were collected. Jaouadi et al. (2019) reported this effect by studying various samples of *T. algeriensis*, grown in different climates ranging from sub-humid to arid, and whose methanolic extracts’ IC_50_ in the β-carotene bleaching assay varied from 0.03 to 1.83 mg/mL, and thus demonstrated the effect of the various edaphoclimatic conditions, which affect each sample’s phytochemical composition and consequently the antioxidant capacity [34]. This fact further intensifies the need to achieve a full phytochemical composition in order to correlate with the potential activities of any medicinal and aromatic plant species.

### 2.4. T. capitellatus Extracts Inhibit Key Enzymes and Show Therapeutic Potential

Altered activity of acetylcholinesterase (AChE) has been described as an hallmark of Alzheimer’s disease [35]. Thus, the in vitro AChE inhibition assay has been widely used to screen for new neuroprotective agents, and among them, various *Thymus* species extracts and their constituents.

In the present research, both AD and HE extracts inhibited AChE activity (Table 3). AD extract inhibited 55.72% at 1 mg/mL, showing an IC_50_ of 0.79 mg/mL, while HE extract performed better, giving an inhibition of 69.28% at 1 mg/mL and an IC_50_ of 0.36 mg/mL. Both extracts showed a dose-dependent inhibition, and the higher content of phenolic compounds in the HE extract partially justifies the higher neuroprotective activity (Table 2 and Table 3). Oleanolic and ursolic acids’ effects on AChE inhibition are well-described [36], and as these are only present in HE extracts, their contribution to *T. capitellatus* HE neuroprotective activity must be taken in account. The values here presented (Table 3) are lower than those reported by Tavares et al. (2011) [16] for HE extracts, where 95% inhibition was achieved at 2 mg/mL of extract, and an IC_50_ of 0.49 mg/mL was observed [16]. As Tavares et al. (2011) [16] did not present a complete phytochemical characterization of the extracts used, the different AChE inhibitions may be due to differences in the extracts’ composition as a result of different edaphoclimatic conditions.

The results reported in the literature cover a wide range of inhibitions, where species such as *T. pulegioides* [17] show promising neuroprotective potential, inhibiting 82–88% of AChE activity at 0.5 mg/mL for both AD and HE extracts, while other species such as *T. praecox* [37] revealed lower inhibition potential, with 9.68% and 14.58% inhibition for aqueous and ethanolic extracts, respectively, at 2 mg/mL. AChE inhibition is certainly correlated to *Thymus* species phytochemical composition, as the modulation of this enzyme activity has been reported for rosmarinic acid [38], present in high concentrations in *T. pulegioides* [17] extracts, and also for flavonoids such as luteolin [35] and quercetin [39].

The tyrosinase inhibition assay is widely used for the screening of natural products’ bioactivities due to its double application, because in addition to skin melanin synthesis, whose inhibition potentializes its use as a skin bleaching agent, the inhibition of neuromelanin synthesis is also of great interest in the search for neuroprotective agents [17]. As seen in Table 3, at 1 mg/mL, *T. capitellatus* extracts inhibited 22.37% (AD) and 28.17% (HE) of tyrosinase activity, showing dose-dependent inhibition when compared to the rate of inhibition at 0.5 mg/mL. AD and HE extracts of *T. pulegioides* [17] presented ~94% inhibition at 0.5 mg/mL. Unlike *T. capitellatus* HE extract, *T. pulegioides* presented residual levels of the pentacyclic triterpenoids oleanolic and ursolic acid, suggesting that these compounds are not responsible for the inhibition observed, and thus it is most likely dependent on rosmarinic acid and hexuronide derivatives of luteolin and eriodyctiol, which are present in high quantities in *T. pulegioides* extracts [17], contrarily to *T. capitellatus* (Table 2).

Regarding the anti-diabetic activity of *T. capitellatus* extracts, this bioactivity is evaluated through colorimetric methods in which the inhibition of α-amylase and α-glucosidase is quantified. The purpose of this assay is to screen for a new functional food, or source of nutraceuticals, capable of reducing the hydrolysis of complex sugars at the intestinal level, and therefore the further absorption of monosaccharides. Although phenolic compounds such as rosmarinic acid, luteolin or quercetin can effectively inhibit both α-amylase and α-glucosidase activity [20,40], *T. capitellatus* extracts present a mild anti-diabetic activity, mostly through the preferential inhibition of α-glucosidase. AD and HE extracts showed dose-dependent inhibition of α-amylase activity, but only inhibited 4.10% and 8.71% of α-amylase activity, respectively, at 1 mg/mL of extract, correlated with the higher concentration of phytochemicals in HE extract. Inhibition of α-glucosidase activity, at both 0.5 mg/mL and 1 mg/mL, was similar in both extracts, revealing an inhibition mechanism not dependent on the presence of terpenoids. The highest inhibition achieved was 24.71% for 1 mg/mL of AD extract; however, this was not statistically different from the inhibition produced by HE extracts (23.02 ± 1.12% inhibition at 1 mg/mL), and both extracts presented dose-dependent inhibition (Table 3).

*T. capitellatus* extracts showed poor capacity to inhibit elastase activity, and only AD extract at 1 mg/mL inhibited 7.16% of elastase activity (Table 3), suggesting that phenolic acids such as rosmarinic acid, salvianolic acids and glycosidic derivatives of common flavonoids do not target this enzyme. These findings suggest that the potential application of *T. capitellatus* extracts in anti-aging products must not be dependent on elastase inhibition, but rather on other bioactivities. Additionally, these results corroborate those reported for *T. pulegioides* extracts, where also only the AD extract presented inhibition (21.43%) [17], although higher than that reported in the current research (Table 3).

### 2.5. Anti-Proliferative Activity of T. capitellatus Extracts

When ingested as herbal teas, infusions or as condiments in foods, the primary point of contact of phytochemicals derived from medicinal and aromatic plants with the organism is the gastrointestinal tract. For this reason, when species such as *T. capitellatus* are screened as potential functional foods, the anti-proliferative/cytotoxic activity towards intestinal cells is commonly addressed. The Caco-2 cell line, although derived from tumoral tissue, presents normal metabolism and protein expression, unlike other intestinal tumoral cell lines, and is a very well-characterized cell model, thus being widely used to access cytotoxicity in the intestinal tract [41,42]. Following intestinal tract absorption, hepatic tissue is the secondary point of contact with the extracts, where metabolization may occur within the first pass effect. To study the interaction of *T. capitellatus* extracts with hepatic tissues, another well-characterized cell line, HepG2, was used. These cells have been shown to maintain typical hepatocyte membrane receptor expression, cytochrome P450-dependent enzymes, glucuronic- and sulphate-conjugation abilities and lipoprotein synthesis [43].

**Figure 3:**
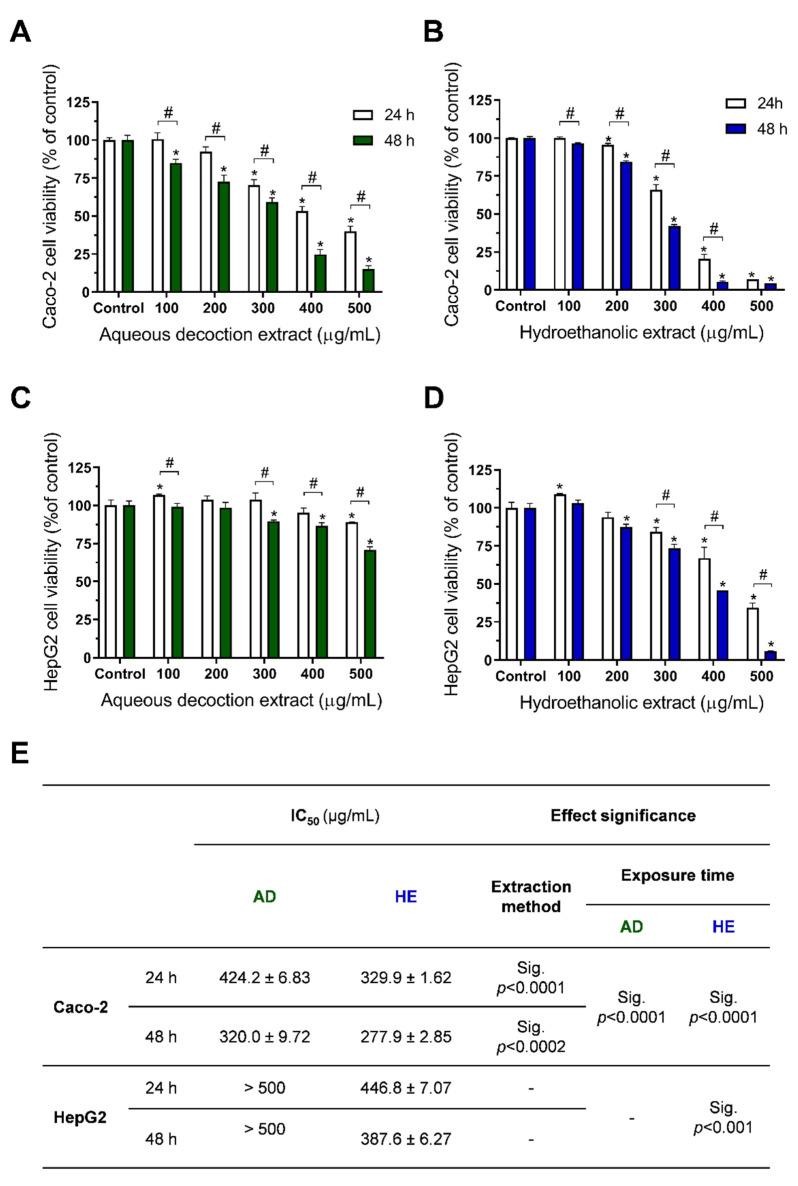
*Thymus capitellatus* aqueous (**A**,**C**) and hydroethanolic (**B**,**D**) extracts’ anti-proliferative/cytotoxic activity against Caco-2 and HepG2 cells, after 24 or 48 h of exposure (as denoted), and (**E**) IC_50_ values obtained from each condition. The results are expressed as mean ± SD, *n* = 4. Significant statistical differences were marked as “*” when between a concentration and the respective control and “#” between the same concentration at different exposure times when *p* < 0.05.

For both cell lines, a dose- and time-dependent cytotoxicity was observed when exposed to *T. capitellatus* HE extracts (Figure 3B,D), with the Caco-2 cells having higher sensitivity to the HE extract action, as the IC_50_ at 24 h exposure was 329.9 µg/mL, lower than that presented for HepG2 at a higher exposure time (48 h) (Figure 3E). Regarding AD extracts, HepG2 cell viability only reduced to 75% of the control at 500 µg/mL. As observed for HE extract, Caco-2 cells were more sensitive to AD extract than HepG2 cells, showing an IC_50_ of 424.4 and 320 µg/mL for 24 h and 48 h exposure, respectively. Additionally, the higher amount in phenolic compounds and in terpenoids observed in HE extracts presents a direct correlation with the anti-proliferative/cytotoxic activity observed, since HE extracts produce lower IC_50_ values than the AD extract, regardless of the cell line or exposure time used (Figure 3E).

This pattern was also observed for Caco-2 and HepG2 cells exposed to other *Thymus* species extracts obtained with the same extraction methods. When compared to the most consumed *Thymus* species worldwide, *T. vulgaris* AD extracts also presented IC_50_ > 500 µg/mL for HepG2, and a slightly lower IC_50_ for Caco-2 cells [19]. However, when exposed to *T. vulgaris* HE extracts for 48 h, Caco-2 cells’ IC_50_ is ~1.38-times higher than that observed under the same conditions for *T. capitellatus*. This could be an effect of both ursolic and oleanolic acid present in higher quantities in *T. capitellatus* HE extract, a conclusion supported by *T. carnosus’* anti-proliferative/cytotoxic activity against the same cell lines, where a composition-dependent effect is observed between AD and HE extracts, mainly due to the presence of high quantities of both terpenoids. Ursolic acid’s concentration in *T. carnosus* HE extract is ~2.59 times higher than that in *T. capitellatus* HE extract, which resulted in a lower IC_50_ value (31.66 µg/mL; 24 h exposure) [7,8]. Additionally, a selective cytotoxicity to Caco-2 cells was observed, when compared to HepG2 cells, due to hepatocytes’ higher capacity to metabolize and eliminate xenobiotic compounds.

In a previous study, using *T. mastichina* AD extract, which is rich in rosmarinic acid, salvianolic acid A isomer, salvianolic acid K, salvianolic acid I and salvianolic acid B/E isomer [18], a high anti-proliferative/cytotoxic effect was observed in Caco-2 (IC_50_ = 95.65 µg/mL) and in HepG2 cells (IC_50_ = 285.03 µg/mL) [18]. However, these cell lines were less sensitive to *T. capitellatus* AD extracts (Figure 3E), which can be justified by the different extract composition (i.e., lower content or absence of some of these compounds).

Nevertheless, the lower cytotoxicity observed for *T. capitellatus* extracts creates a window of opportunity to exploit its potential as a functional food. Analyzing the antioxidant potential through the in vitro methodologies here reported, we observed that for example in nitric oxide or superoxide radical scavenging, at the highest concentration tested (1 mg/mL), the final concentration of the extract when in the reaction mixture was 100 and 30 µg/mL, respectively, both concentrations that did not induce cytotoxicity, independently of the cell line, extract or exposure time. For the β-carotene bleaching assay, the IC_50_ values correspond to a final concentration of 21.66 and 25 µg/mL, respectively, also well above cytotoxic concentrations, and therefore unveiling the potential of *T. capitellatus* extracts as dietary antioxidant agents, which should be further confirmed through in vitro cell-based assays.

Concerning potential bioactivities in cell models, the main compounds of *T. capitellatus* extracts were screened for antioxidant and anti-inflammatory activity, biological processes in which radicals (such as nitric oxide, superoxide, hydroxyl) and lipid peroxidation products play significant roles.

Rosmarinic acid was reported to possess anti-inflammatory activity through the inhibition of nitric oxide production [7]. Additionally, anti-inflammatory and antioxidant activities of quercetin and some of its derivatives have also been described [44], which is particularly relevant given the high number of quercetin derivatives present in *T. capitellatus* extracts (Table 2). Luteolin and ursolic acid were found to actively reduce oxidative DNA damage and induce DNA repair in Caco-2 cells [45], and luteolin-7-*O*-glucoside is able to modulate Nrf2/MAPK (erythroid 2-related factor 2/mitogen-activated protein kinases) pathway and HO-1 (heme-oxygenase-1) induction [46], as well as being able to modulate the NF-κB (factor nuclear kappa B), AP-1 (activator protein 1) and PI3K-AKT (phosphatidylinositol-3-kinase—protein kinase B) pathways in a lipopolysaccharide (LPS)-induced macrophage cell model, unveiling the mechanism of action for its antioxidant and anti-inflammatory activity [46]. Therefore, with these phytochemicals being the main components of *T. capitellatus* extracts, potential antioxidant and anti-inflammatory activity may be achieved, by either direct radical scavenging or metabolic pathways modulation, further increasing the value of and interest in this near-threatened species.

Additionally, *T. capitellatus* HE extract IC_50_ for AChE inhibition was 0.36 mg/mL (Table 3), which represents a final in-well concentration of 79.92 µg/mL, the tyrosinase inhibition by *T. capitellatus* HE extract was 17.58% when tested at 0.5 mg/mL (final in-well concentration of 135 µg/mL), and anti-diabetic activity was reported at 52.63 µg/mL (1 mg/mL; α-amylase) and 125 µg/mL (0.5 mg/mL; α-glucosidase), therefore proving that *T. capitellatus* extracts can potentially provide neuroprotection at non-cytotoxic concentrations, dependent on the phytochemicals’ absorption and metabolization that should be further assessed, and exhibit mild anti-diabetic activity, as the enzymes’ inhibition is performed in the intestinal lumen without the need to be absorbed and at concentrations that do not compromise the intestinal tract.

## 3. Materials and Methods

### 3.1. Standards and Reagents

Commercial standards of salvianolic acid A, rosmarinic acid, quercetin-3-*O*-glucoside and ursolic acid were purchased from Sigma-Aldrich/Merck (Algés, Portugal). Caffeic acid, luteolin-7-*O*-hexoside and eriodyctiol-7-*O*-hexoside were obtained from Extrasynthese^®^ (Genay, France). Oleanolic acid was obtained from Santa Cruz Biotechnology Inc. (Frilabo, Porto, Portugal). Dulbecco’s modified Eagle medium (DMEM), sodium pyruvate, penicillin, streptomycin, versene, L-glutamine, trypsin-EDTA, and fetal bovine serum (FBS) were obtained from Gibco (Alfagene, Lisboa, Portugal). Alamar Blue^®^ was obtained from Invitrogen, Life-Technologies (Alfagene, Lisboa, Portugal). Methanol, ethanol, formic acid, and acetic acid were HPLC- or MS-grade, according to the analysis, and were purchased from Sigma-Aldrich/Merck (Algés, Portugal). β-Carotene, linoleic acid, sodium nitrite, 2,2-azino-bis (3-ethylbenzothiazoline-6-sulfonic acid) diammonium salt (ABTS), (±)-6-hydroxy-2,5,7,8-tetramethylchromane-2-carboxylic acid (Trolox), xanthine oxidase, sodium nitroprusside, sulfanilamide, *N*-(1-naphthyl)ethylenediamine dihydrochloride, potassium persulfate, Folin–Ciocalteu’s reagent, ethylenediaminetetraacetic acid (EDTA), ascorbic acid, hypoxanthine, aluminium chloride (III), sodium molybdate, trichloroacetic acid (TCA), thiobarbituric acid (TBA), nitro blue tetrazolium, 2-deoxy-D-ribose, hydrogen peroxide (30% solution) and all the enzymes and reagents for the enzymatic assays were obtained from Sigma-Aldrich/Merck (Algés, Portugal). Other salts and reagents not mentioned were obtained from Sigma-Aldrich/Merck (Algés, Portugal).

### 3.2. Plant Material

*T. capitellatus* Hoffmanns & Link’s aerial parts (leaves and stems) were collected in Arrábida National Park (Sesimbra, Setúbal, Portugal). The harvest was authorized by the Institute for the Conservation of Nature and Forests (ICNF, I.P.; Portugal) (License n. 867/2018/RECOLHA and 868/2018/RECOLHA). A portion of plant material containing leaves and stems was used for authentication by the Botanical Garden office at the University of Trás-os-Montes and Alto Douro (UTAD, Vila Real, Portugal), providing the voucher specimen n. HVR22497. After harvest, the plant material was prepared for lyophilization (Dura Dry TM μP freeze-drier; −45 °C and 250 mTorr) by rinsing the samples with distilled water, weighing, and freezing. Once lyophilized, the plant material was ground and properly stored (cool and dry place, protected from light) until further extraction and analysis.

### 3.3. Preparation of Extracts

Two type of extracts were obtained from the dried and ground *T. capitellatus* plant material: aqueous decoction (AD) and exhaustive hydroethanolic (HE) extraction, as described by Martins-Gomes et al. (2018) [7]. AD extraction method was used due to its resemblance to procedures used in human consumption (e.g., as a condiment or as an infusion). Briefly, 500 mg of dried plant material was added to 150 mL of distilled water, the mixture was heated to 100 °C, and kept boiling for 20 min, under agitation, after which it was left to cool at room temperature. The mixture was then filtered twice (Whatman n. 4 filter for larger particles followed by a fiberglass filter (1.2 µm pore size); acquired from VWR International Ltd., Alfragide, Portugal), and concentrated to 100 mL in a rotary evaporator (35 °C) [7].

Regarding HE exhaustive extraction, Martins-Gomes et al. (2018) reported the efficiency of this method to obtain all of the extractable phenolic compounds within the plant material when repeating the procedure thrice. Briefly, to 500 mg of dried plant material 50 mL of an ethanol:water solution (80:20, *v*/*v*) was added, and the mixture was agitated (orbital shaker, 150 rpm) for one hour, followed by a centrifugation (7000 rpm, Sigma Centrifuges 3–30 K, St. Louis, MO, USA). The supernatant was collected, and the pellet was used to repeat the same procedure two more times. The three supernatants were combined and filtered as described for the AD method. The ethanol in the mixture was removed, while concentrating the extract in a rotary evaporator [7]. For both extraction methods, the procedure was repeated three times. All extracts were frozen, lyophilized and weighed to calculate the yields, and were properly stored until further analysis.

### 3.4. Total Phenolic Compounds, Ortho-Diphenols and Total Flavonoids Content

The total phenolic compound content (TPC) was established using the Folin–Ciocalteu method as described by Machado et al. (2013) [47]. To 1 mL of *T. capitellatus* extract (0.5 mg/mL), 0.5 mL of Folin–Ciocalteu reagent was added, followed by 1 mL of 7.5% sodium carbonate (Na_2_CO_3_) and 7.5 mL of distilled water. After 60 min incubation at room temperature, the absorbance was read at 725 nm using a spectrophotometer (PerkinElmer, Lambda 25 UV/VIS Spectrometer). TPC was expressed as caffeic acid equivalents (mg CA eq./g lyophilized plant or mg CA eq./g extract). For the literature comparison in the discussion section, TPC results were also expressed in gallic acid equivalents (mg GAE/g lyophilized plant), converted based on gallic acid/caffeic acid calibration curves relative slopes.

The *ortho*-diphenols content (ODC) was determined using the sodium molybdate colorimetric method, as described by Taghouti et al. (2019) [18]. To 4 mL of *T. capitellatus* extract (0.1 mg/mL), 1 mL of 5% sodium molybdate (Na_2_MoO_4_) was added. After 15 min incubation at room temperature, the absorbance was measured at 370 nm. The ODC content was expressed as caffeic acid equivalents (mg CA eq./g lyophilized plant or mg CA eq./g of extract).

The total flavonoid content (TFC) assay was performed using the aluminum chloride colorimetric method, as described by Taghouti et al. (2019) [18]. To 1 mL of *T. capitellatus* extract (0.5 mg/mL), 150 μL of 5% of sodium nitrite (NaNO_2_) was added, followed by 5 min incubation at room temperature. Then, 150 μL of 10% aluminium chloride (AlCl_3_) solution was added, the mixture was allowed to incubate for 6 min, and 1 mL of 1 M sodium hydroxide (NaOH) was added to finalize the reaction. The absorbance was read at 510 nm. TFC was expressed as catechin equivalents (mg C eq./g lyophilized plant or mg C eq./g extract). TPC, ODC and TFC analyses were performed in triplicate (*n* = 3).

### 3.5. Profiling and Quantification of Individual Phenolic Compounds by HPLC-DAD and HPLC-ESI-MS^n^

The RP-HPLC-DAD analysis was performed using a Thermo Fisher Scientific Vanquish Core HPLC system (Waltham, MA, USA) equipped with a pump, column compartment, auto-sampler, and diode array detector. Chromatographic separation was performed using a C18 column (Merck Purospher^®^ STAR, Hibar^®^ C18; 250 mm × 4.6 mm; particle size 5 μm) using 0.1% formic acid and methanol as solvents A and B, respectively, with an injection volume of 100 μL, the temperature was kept at 40 °C, and the flow rate was 0.5 mL/min. The initial gradient was 90% A/10% B, maintained for 10 min of run preparation, after which the injection was performed. Solvent B’s percentage was increased from 10% to 30% in 15 min, followed by a second increase to 56% in 45 min. Solvent B’s percentage was then raised to 100% in 5 min as a cleaning step, after which it was returned to the initial gradient, for a total time of 75 min. UV/Vis detection was performed at 200–600 nm. Chromeleon software (Version 7.3; Dionex, Sunnyvale, CA, USA) was used for data acquisition, peak integration, and data analysis.

RP-HPLC-ESI-MS^n^ analysis was performed as previously described by Martins-Gomes et al. (2018) [7], using a Thermo Scientific system equipped with a Finnigan Surveyor Plus auto-sampler, pump, photodiode array detector, and an LXQ Linear ion trap detector. Electrospray ionization (ESI) was performed in negative mode (spray voltage: −4 kV; capillary voltage: −5 kV; capillary temperature: 350 °C). Chromatographic separation was performed with a Luna C18 column (250 mm × 4.6 mm, 5 μm; Phenomenex (Aschaffenburg, Germany)). Program conditions, flow rate, solvents, temperature, injection volume and detection parameters were used exactly as described by Martins-Gomes et al. (2018) [7].

The identification of individual phenolic compounds present in *T. capitellatus* extracts was based on UV-VIS spectra, mass spectra and retention time obtained for the extracts and comparison with commercial standards and/or literature data. The quantification of individual phenolic compounds was performed using calibration curves of commercial standards, when available, or using the aglycones or standard compounds with structural similarity. Luteolin, luteolin derivatives, apigenin derivatives and chrysoeriol derivatives were quantified as luteolin-7-*O*-glucoside (PubChem CID: 5280637); eriodyctiol derivatives were quantified as eriodyctiol-7-*O*-glucoside (Pubchem CID 13254473); quercetin derivatives were quantified as quercetin-3-*O*-glucoside (Pubchem CID 2520336); rosmarinic acid, salvianolic acid A isomer and salvianolic acids K and K isomer were quantified as rosmarinic acid (PubChem CID: 5281792). Caffeic acid (PubChem CID: 689043), oleanolic acid (PubChem CID: 10494) and ursolic acid (PubChem CID: 64945) were quantified with their respective standards. HPLC-DAD and HPLC-ESI-MS^n^ analysis TPC, ODC and TFC analysis were performed in triplicates (*n* = 3).

### 3.6. Quantification of Oleanolic and Ursolic Acids in Hydroethanolic Extracts

Ursolic (UA) and oleanolic acids (OA) were detected and quantified in *T. capitellatus* HE extracts by RP-HPLC-DAD. The chromatographic separation was performed using the same equipment, column, solvents, temperature and injection volume described above. The chromatographic separation was achieved with an initial gradient of 20% A/80% B. Solvent B’s percentage was increased to 90% in 45 min, and held isocratically for 9 min, after which the initial gradient was restored, for a total run time of 55 min. UV/Vis detection was performed at 210 nm. Ursolic and oleanolic acid identification and quantification were performed using spectra comparison and calibration curves of commercial standards, respectively. HPLC-DAD analysis was performed in triplicate (*n* = 3).

### 3.7. In Vitro Antioxidant Activity Assessment

#### 3.7.1. ABTS Radical Cation (ABTS^+•^) Scavenging Assay

The ABTS^•+^ scavenging assay was performed as described by Taghouti et al. (2018) [17]. Briefly, ABTS^•+^ was generated by reacting equal volumes of ABTS solution (2,2-azino-bis(3-ethylbenzothiazoline-6-sulfonic acid) diammonium salt; 7 mM in water) and potassium persulfate (K_2_S_2_O_8_; 2.45 mM in water) for 15–16 h (in the dark, at room temperature). After incubation, the concentrated radical solution was diluted using acetate buffer (20 mM, pH 4.5) in order to obtain an absorbance of 0.700 ± 0.02 (at 734 nm). *T. capitellatus* extracts’ scavenging capacity was evaluated by adding 200 μL of the extract (0.1 mg/mL) to 2 mL of the diluted ABTS^•+^ solution. After 15 min of incubation, the absorbance was read at 734 nm. Trolox ((±)-6-hydroxy-2,5,7,8-tetramethylchromane-2-carboxylic acid) was used as a standard antioxidant. Results were expressed as Trolox equivalents (mmol Trolox/g lyophilized plant or mmol Trolox/g extract). The assay was performed in triplicate (*n* = 3).

#### 3.7.2. Hydroxyl Radicals Scavenging Assay

Hydroxyl radical (^•^OH) scavenging activity was determined as described by Taghouti et al. (2019) [18]. Briefly, to 500 μL of *T. capitellatus* AD extract (0.1–1 mg/mL) (HE extracts were not tested due to ethanol interference) was added 100 μL of each of the following: deoxyribose (20 mM), ascorbic acid (1 mM), iron (II) chloride (FeCl_2_; 1 mM), hydrogen peroxide (H_2_O_2_; 10 mM), and 400 μL of phosphate buffer solution (20 mM; pH 7.4). Another set of samples were prepared with the same reaction mixture described above, but with the addition of 100 μL of EDTA solution (1 mM), and both sets were incubated for 60 min at 37 °C. After incubation, 1.5 mL of 0.5% TBA (prepared in 10% TCA) was added, followed by 15 min incubation at 100 °C. Absorbance was read at 532 nm. For control, the same reaction mixture and procedure were used (both with and without EDTA), and distilled water replaced the extract solutions. The assay was performed in triplicate (*n* = 3). Radical scavenging activity was expressed as the percentage of inhibition using Equation (1):

(1) $$\mathrm{Inhibition}\;\left(\%\right)=\frac{\mathrm{Blank}\;\mathrm{abs}-\mathrm{Sample}\;\mathrm{abs}\;}{\mathrm{Blank}\;\mathrm{abs}\;}\times 100$$

#### 3.7.3. Nitric Oxide Radical Scavenging Assay

Nitric oxide radical (NO^•^) scavenging activity was performed as described by Sreejayan and Rao (1997) [48], adapted to 96-well microplates. Sodium nitroprusside solution (Na_2_[Fe(CN)_5_NO]; 5 mM prepared in phosphate buffer (0.1 M H_3_PO_4_; pH 7.4)) was purged with air, to oxygenate the solution, for 15 min under agitation. To 20 μL of *T. capitellatus* AD extract (0.1–1 mg/mL) (HE extracts were not tested due to insolubility in water and due to ethanol interference with the method), 180 μL of sodium nitroprusside solution was added, followed by 120 min incubation at 35 °C under a light source. Griess reagent (equal volumes of 1% sulfanilamide (in 5% H_3_PO_4_) and 0.1% *N*-alpha-naphthyl-ethylenediamine (in water)) was used to quantify NO^•^. To 100 μL of the reaction mixture (extracts and sodium nitroprusside solution) an equal volume of Griess reagent was added and the absorbance was measured at 545 nm after 5 min of incubation (Multiskan EX microplate reader (MTX Labsystems; Bradenton, FL, USA)). As the negative control, using the same reaction mixture and procedure as described above, distilled water was used to replace the extract solutions, and sodium nitrite was used as the positive control. NO^•^ scavenging was calculated according to Equation (1) and expressed as the inhibition percentage. The assay was performed in triplicate (*n* =3).

#### 3.7.4. Superoxide Radical (O2^•−^) Scavenging Assay

Scavenging of O_2_^•−^ by *T. capitellatus* extracts was performed as described by Tao et al. (2014) [49] with modifications. Briefly, to 6.7 µL of extracts (0.1–1 mg/mL) was added 193.3 µL of a reaction solution containing 6.43 µL of 4 mM hypoxanthine, 12.86 µL of 4 mM NBT (nitro blue tetrazolium) and 174 µL of 50 mM phosphate buffer (pH 8). After 2 min incubation at 37 °C, the reaction was initiated by adding 20 µL of 0.04 U/mL xanthine oxidase solution (in 50 mM phosphate buffer (pH 8) with 0.5 mM EDTA). The absorbance was measured at 570 nm (Multiskan EX microplate reader (MTX Labsystems; Bradenton, FL, USA)), immediately after the enzyme addition (blank), and then samples were incubated for 20 min at 37 °C, after which the reaction was stopped by adding 20 µL of 0.6 M HCl. Absorbance was measured again at 570 nm and the results were expressed as the percentage of inhibition against the negative control (H_2_O instead of sample). DMSO (10%, in water) was used to dissolve hydroethanolic extracts, as it was previously tested to assure no interference with this assay. The assay was performed in triplicate (*n* = 3).

#### 3.7.5. β-Carotene Bleaching Assay

The β-carotene bleaching assay was performed as described by Afonso et al. (2017) [32] with modifications. Briefly, in a round-bottomed evaporation flask were mixed 0.25 mL of β-carotene solution (2 mg/mL; in chloroform) and 500 mg of Tween 20. Chloroform was evaporated in a rotary evaporator (35 °C) and then 25 mg of linoleic acid was added, followed by 50 mL of distilled water. Using the rotation motion of the rotary evaporator (no vacuum and no temperature), the mixture was gently homogenized to produce the emulsion. To 50 µL of the extract solution (0.1–1 mg/mL) 250 µL of the emulsion was added, the absorbance was immediately measured at 450 nm (blank), and then the mixture was incubated for 120 min at 50 °C. Immediately after incubation, the 96-well plate was placed on ice to stop the reaction and the absorbance was measured at 450 nm. Trolox was used as a standard antioxidant. DMSO (10%; in water) was used as a control for hydroethanolic extracts and presented no inhibition. Results were expressed as % of inhibition and calculated as reported by Afonso et al. (2017). The assay was performed in triplicate (*n* = 3).

### 3.8. In Vitro Enzymatic Inhibition Assays

Aqueous decoction and hydroethanolic extracts of *T. capitellatus* were evaluated for their capacity to inhibit target enzymes involved in neuroprotection (acetylcholinesterase and tyrosinase), anti-aging (tyrosinase and elastase) and anti-diabetic (α-amylase and α-glucosidase) activities using the methodologies described in Taghouti et al. (2018) [17].

For acetylcholinesterase (AChE) inhibition, to 50 µL of various concentrations of each extract (0.1 to 1 mg/mL), 125 µL DTNB (0.3 mM; in 50 mM Tris-buffer, pH 8) and 25 µL of acetylthiocholine iodide (1.5 mM; in water) were added, followed by 2 min incubation. After incubation, 25 µL of AChE (0.026 U/mL; in 20 mM Tris-HCl buffer, pH 7.5) was added, and the samples were incubated for 10 min at room temperature. Absorbance was measured at 405 nm and results were expressed as the inhibition percentage.

Tyrosinase inhibition was performed by adding 25 µL of the extract (concentration range 0.1 to 1 mg/mL) to 80 µL of phosphate buffer (50 mM, pH 6.8) and 40 µL of L-DOPA (2.5 mM; in water). After 2 min incubation at 37 °C, the reaction was initiated by adding 40 µL of tyrosinase (40 U/mL; in 50 mM phosphate buffer, pH 6.5). Absorbance was measured at 492 nm after 10 min incubation at 37 °C, and the results were expressed as the inhibition percentage.

For the elastase inhibition assay, 50 μL of each extract (concentration range 0.1 to 1 mg/mL) was added to 160 μL of Tris-HCl buffer (0.2 M, pH 8.0) and 20 μL of *N*-(methoxysuccinyl)-ala-ala-pro-val-4-nitroanilide (0.8 mM; in Tris-HCl buffer). After 10 min of incubation, the reaction was initiated by adding elastase solution (20 μL; 0.4 U/mL, in Tris-HCl buffer), followed by a second incubation of 20 min. The absorbance was then measured at 410 nm and results were expressed as the inhibition percentage.

For the α-amylase inhibition assay, to 5 µL of extract (concentration range 0.1 to 1 mg/mL) were added 35 µL of both PBS and starch (0.05%; prepared in water), and the mixture was left to incubate for 2 min at 37 °C. The reaction was initiated by the addition of 20 µL of alpha-amylase solution (50 μg/mL; in 10 mM phosphate buffer, pH 6.9), incubated for 10 min at 37 °C, and then stopped by the addition of 50 µL of 0.1 M HCl. Lugol solution was prepared according to Taghouti et al. (2018) and added (150 µL) to the mixture. Absorbance was measured at 580 nm and acarbose was used as the positive control. The results were expressed as the inhibition percentage.

For the α-glucosidase inhibition assay, a rat’s intestinal acetone powder solution was prepared as described by Taghouti et al. (2018), and 100 µL of the prepared solution was added to 50 µL of the extract (concentration range 0.1 to 1 mg/mL), followed by 10 min incubation at room temperature. The reaction was initiated with the addition of 50 µL of *p*-nitrophenyl-α-D-glucopyranoside (5 mM; in sodium phosphate buffer). After 30 min incubation at 37 °C, absorbance was measured at 405 nm, and acarbose was used as the positive control. The results were expressed as the inhibition percentage. All enzymatic assays were performed in triplicate (*n* = 3).

### 3.9. Cell Cultures and In Vitro Cell Viability Assay

The anti-proliferative/cytotoxic activity of *T. capitellatus* AD and HE extracts was evaluated in two human cell lines: Caco-2 (human colon adenocarcinoma cell line; CLS, Cell Lines Service, Eppelheim, Germany) and HepG2 (human hepatocellular carcinoma cell line; ATCC^®^ Number: HB-8065TM).

Cell culture maintenance and handling was performed as described by Silva et al. (2020) [3]. Briefly, both cell lines were maintained at 37 °C in 5% CO_2_/95% air with controlled humidity and were cultured in Dulbecco’s modified Eagle medium (DMEM), supplemented with 10% fetal bovine serum (FBS), 1 mM L-glutamine and antibiotics (100 U/mL penicillin and 100 μg/mL streptomycin). For the assays, cells were seeded in 96-well plates (at 5 × 10^4^ cells/mL; 100 μL/well) and allowed to adhere and stabilize for 48 h prior to the assay.

Stock solutions (10 mg/mL) of *T. capitellatus* AD and HE extracts were prepared in PBS and in 10% DMSO (prepared in PBS), respectively. The DMSO final concentration in the test solutions did not exceed 1%.

The anti-proliferative/cytotoxic effect of extracts was evaluated using Alamar Blue^®^ assay as described by Andreani et al. (2014) [50]. Briefly, the culture medium was replaced by extract solutions (100 μL/well), consisting of dilutions of the respective stock solutions in FBS-free culture medium (extract solutions range 100–500 μg/mL). Cells were incubated with test solutions for 24 h and 48 h in independent assays, after which the test solutions were removed and replaced by 100 μL of 10% Alamar Blue^®^ solution (in FBS-free culture medium). After 5 h incubation, the absorbance was read at 570 nm and 620 nm (Multiskan EX microplate reader (MTX Labsystems; Bradenton, FL, USA)). The control (non-exposed cells) was performed in each assay and results were calculated as described by Andreani et al. (2014) [50], and expressed as cell viability (% of control), from 3 independent assays, each performed in quadruplicate.

### 3.10. Data and Statistical Analysis

The results are presented as mean ± SD. The IC_50_ values for cell-based assays were calculated as described by Silva et al. (2019) [51]. Analyses of variance (ANOVA) followed by Tukey’s multiple test were performed to analyze statistically significant differences. Correlations were evaluated using Pearson’s coefficient (significant if *p* < 0.05). Statistical analyses and graphic design were performed using GraphPad Prism version 8 (GraphPad Software Inc., San Diego, CA, USA) and Microsoft Office Excel (Microsoft Corporation, Washington, DC, USA).

## 4. Conclusions

In the present work, the phytochemical composition of aqueous and hydroethanolic extracts of *T. capitellatus* is fully described for the first time. In addition to common phenolic acids of the *Thymus* genus, such as caffeic and rosmarinic acid, the extracts present a high content of flavonoids, with several glycoside derivatives of luteolin, quercetin, apigenin and eriodictyol. Contrarily to other *Thymus* species, where rosmarinic acid appears as the main component, quercetin-(?)-*O*-hexoside and oleanolic acid appear as the most abundant components, unveiling a unique phytochemical composition. In addition, *T. capitellatus* extracts present good antioxidant potential against nitric oxide, hydroxyl and superoxide radicals, as well as high inhibition of lipid peroxidation. As well as being capable of exerting anti-proliferative activity in intestinal and hepatic tumoral cell lines, it is reported in the present research that *T. capitellatus* extracts present a high potential for antioxidant, neuroprotective and anti-diabetic activities at non-cytotoxic concentrations, thus offering a promising alternative as a safe functional food and source of nutraceuticals.

## Figures and Tables

**Table 1:** Extraction yields, chemical composition and ABTS radical scavenging activity of *Thymus capitellatus* extracts.

		*Thymus capitellatus*		
		AD	HE	E.M.E.
Extraction yield (%, *w*/*w*)		15.82 ± 2.30	16.84 ± 2.43	n.s.
		Chemical composition		
Total phenols content (mg caffeic acid equivalent/g)	Ext.	150.29 ± 5.29	140.81 ± 5.42	n.s.
	D.P.	23.77 ± 0.84	23.72 ± 0.91	n.s.
*Ortho*-diphenols content (mg caffeic acid equivalent/g)	Ext.	134.01 ± 5.08	146.02 ± 3.62	*
	D.P.	21.36 ± 0.80	24.60 ± 0.61	*
Total flavonoids content (mg catechin equivalent/g)	Ext.	174.26 ± 11.58	188.84 ± 7.81	n.s.
	D.P.	27.56 ± 1.83	31.81 ± 1.31	*

Abbreviations: AD: Aqueous decoction and HE: hydroethanolic extractions; E.M.E.: extraction method effect; equivalent/g. Ext.: mg/g extract. D.P.: mg/g dry plant; n.s.—not significant. For the antioxidant activity assays, the percentage of inhibition obtained for at 1 mg/mL of extract is presented. Tukey’s post hoc test, statistically significant differences (*) between extraction methods if *p* < 0.05. Results are presented as mean ± standard deviation (*n* = 3).

**Table 2:** Phytochemical composition of *T. capitellatus* hydroethanolic (HE) and aqueous decoction (AD) extracts obtained by HPLC/DAD-ESI/MS^n^ analysis.

	Compound	R.T. (min)	ESI-MS2	Quantification				
				HE		AD		E.M.E. Sig.
				mg/g D.P.	mg/g Extract	mg/g D.P.	mg/g Extract	
**1**	Unknown	21.73 ± 0.11	[459]:161	n.q.	n.q.	n.q.	n.q.	
**2**	Unknown	23.13 ± 0.08	[509]:463;441;329;295	n.q.	n.q.	n.q.	n.q.	
**3**	Apigenin-(6,8)-*C*-diglucoside	23.19 ± 0.08	[593]:575;503;473;383;353	n.q.	n.q.	n.q.	n.q.	
**4**	Unknown	23.48 ± 0.02	[455]:409;387;317;233;173	n.q.	n.q.	n.q.	n.q.	
**5**	Hydroxyjasmonic acid-(?)-*O*-hexoside	23.44 ± 0.11	[387]:369;225;207;163	n.q.	n.q.	n.q.	n.q.	
**6**	Caffeic acid	23.76 ± 0.08	[179]:135	0.25 ± 0.02	1.49 ± 0.13	0.63 ± 0.12	3.99 ± 0.73	*
**7**	Eriodictyol-(?)-*O*-hexoside	24.20 ± 0.14	[449]:287	0.49 ± 0.06	2.9 ± 0.38	0.25 ± 0.14	1.61 ± 0.9	*
**8**	Unknown	24.27 ± 0.09	[495]:427;341;333;315	n.q.	n.q.	n.q.	n.q.	
**9**	Eriodictyol-(?)-*O*-hexoside	27.16 ± 0.21	[449]:287	0.84 ± 0.1	4.99 ± 0.61	0.38 ± 0.12	2.43 ± 0.79	*
**10**	Quercetin-(?)-*O*-hexoside	27.33 ± 0.22	[463]:301	5.91 ± 0.68	35.11 ± 4.02	0.67 ± 0.44	4.23 ± 2.78	*
**11**	Luteolin-(?)-*O*-hexoside	29.28 ± 0.12	[447]:285	0.55 ± 0.06	3.26 ± 0.35	0.16 ± 0.07	1.01 ± 0.42	*
**12**	Luteolin-(?)-*O*-hexoside	31.17 ± 0.18	[447]:285	3.94 ± 0.44	23.38 ± 2.6	0.42 ± 0.13	2.63 ± 0.84	*
**13**	Salvianolic acid A isomer	33.84 ± 0.19	[493]:383;313;295	0.32 ± 0.04	1.92 ± 0.24	0.25 ± 0.04	1.6 ± 0.26	*
**14**	Luteolin-(?)-*O*-hexuronide	33.87 ± 0.24	[461]:285	n.q.	n.q.	n.q.	n.q.	
**15**	Quercetin-(?)-*O*-(caffeoyl)-hexoside	33.96 ± 0.25	[625]:463;323;301	1.7 ± 0.08	10.07 ± 0.49	0.56 ± 0.28	3.51 ± 1.76	*
**16**	Chrysoeriol-(?)-*O*-hexoside	34.83 ± 0.08	[461]:299	n.q.	n.q.	n.q.	n.q.	
**17**	Rosmarinic acid	35.19 ± 0.04	[359]:223;179;161	3.66 ± 0.34	21.71 ± 2.03	1.01 ± 0.61	6.37 ± 3.84	*
**18**	Apigenin-(?)-*O*-hexoside	35.79 ± 0.06	[431]:269	0.12 ± 0.02	0.7 ± 0.13	0.1 ± 0.03	0.61 ± 0.19	n.s.
**19**	Acetyl-luteolin-(?)-*O*-hexoside-pentoside	36.09 ± 0.40	[621]:579;561;447;327;285	0.44 ± 0.02	2.59 ± 0.13	0.84 ± 0.17	5.33 ± 1.1	*
**20**	Unknown	36.37 ± 0.05	[549]:531;489;387;207;161	n.q.	n.q.	n.q.	n.q.	
**21**	Salvianolic acid K	36.95 ± 0.10	[555]:537;493;359	0.83 ± 0.06	4.91 ± 0.37	0.64 ± 0.2	4.06 ± 1.25	n.s.
**22**	Quercetin -(?)-*O*-hexoside-deoxy-hexoside	38.17 ± 0.05	[609]:463;301	0.28 ± 0.03	1.64 ± 0.15	0.08 ± 0.03	0.51 ± 0.21	*
**23**	Quercetin-(?)-*O*-hexoside-hexuronide	39.07 ± 0.38	[639]:301	1.03 ± 0.05	6.09 ± 0.31	0.4 ± 0.14	2.51 ± 0.91	*
**24**	Salvianolic acid K isomer	40.76 ± 0.14	[555]:493;359	0.16 ± 0.01	0.93 ± 0.03	0.03 ± 0.01	0.16 ± 0.05	*
**25**	Luteolin-(?)-*O*-hexoside-hexoside	43.06 ± 0.25	[609]:447;323;285	0.85 ± 0.09	5.06 ± 0.53	0.22 ± 0.03	1.36 ± 0.2	*
**26**	Chrysoeriol-(?)-*O*-hexoside-hexoside	43.10 ± 0.15	[623]:461;323;299;285	0.33 ± 0.03	1.94 ± 0.19	0.23 ± 0.05	1.48 ± 0.32	n.s.
**27**	Luteolin	49.05 ± 0.23	[285]:241;217;199;75;151	0.65 ± 0.11	3.85 ± 0.66	0.42 ± 0.15	2.67 ± 0.93	n.s.
**28**	Oleanolic acid			6.02 ± 1.35	35.77 ± 8.04	n.d.	n.d.	*
**29**	Ursolic acid			4.88 ± 1.07	28.97 ± 6.38	n.d.	n.d.	*
		Total phenolic compounds		23.07 ± 1.48	137.67 ± 8.77	7.84 ± 2.14	49.55 ± 13.51	*
			Total flavonoids	17.68 ± 1.17	104.97 ± 6.95	5.19 ± 1.33	32.8 ± 8.42	*
			Total phenolic acids	5.21 ± 0.44	30.96 ± 2.63	2.65 ± 0.89	16.75 ± 5.64	*
			Total terpenoids	10.90 ± 2.43	64.74 ± 14.4	n.d.	n.d.	*

Abbreviations: AD: Aqueous decoction and HE: hydroethanolic extractions; E.M.E.: extraction method effect; equivalent; RT: retention time; ESI-MS^2^: Fragment ions obtained after fragmentation of the pseudo-molecular ion [M]^−^; n.q.: detected but not quantified; n.d.: not detected; n.s.: not significant. Tukey’s post hoc test. Statistically significant differences (*) between extraction methods for mg/g of dry plant (D.P.) if *p* < 0.05. Results are presented as mean ± standard deviation. *n* = 3.

**Table 3:** Assessment of *T. capitellatus* extract’s inhibition of acetylcholinesterase, tyrosinase, elastase, α-amylase and α-glucosidase.

		*Thymus capitellatus* Extracts (mg/mL)		
		AD	HE	E.M.E.
Acetylcholinesterase	IC_50_	0.79 ± 0.05	0.36 ± 0.04	*
	0.5 mg/mL	34.03 ± 3.83	54.22 ± 6.84	*
	1 mg/mL	55.72 ± 6.39	69.28 ± 10.22	n.s.
Tyrosinase	0.5 mg/mL	16.56 ± 4.26	17.58 ± 0.33	n.s.
	1 mg/mL	22.37 ± 1.29	28.17 ± 1.73	*
Elastase	0.5 mg/mL	-	-	n.s.
	1 mg/mL	7.16 ± 1.50	-	*
α-Amylase	0.5 mg/mL	3.17 ± 0.5	4.92 ± 0.19	*
	1 mg/mL	4.10 ± 0.18	8.71 ± 0.59	*
α-Glucosidase	0.5 mg/mL	16.74 ± 1.01	13.30 ± 1.4	*
	1 mg/mL	24.57 ± 0.24	23.02 ± 1.12	n.s

Abbreviations: AD: Aqueous decoction; HE: hydroethanolic extractions; n.s.: not significant; E.M.E.: extraction method effect. Tukey’s post hoc test. Statistically significant differences (*) between extraction methods for mg/g of dry plant (D.P.) if (*p* < 0.05). Results are presented as mean ± standard deviation. *n* = 3.

## Data Availability

Not applicable.

## References

[1] Granato D., Nunes D.S., Barba F.J. (2017). An integrated strategy between food chemistry, biology, nutrition, pharmacology, and statistics in the development of functional foods: A proposal. Trends Food Sci. Technol..

[2] Nieto G. (2020). A Review on Applications and Uses of *Thymus* in the Food Industry. Plants.

[3] Silva A.M., Martins-Gomes C., Souto E.B., Schäfer J., Santos J.A., Bunzel M., Nunes F.M. (2020). *Thymus zygis* subsp. zygis an Endemic Portuguese Plant: Phytochemical Profiling, Antioxidant, Anti-Proliferative and Anti-Inflammatory Activities. Antioxidants.

[4] Lorenzo J.M., Mousavi Khaneghah A., Gavahian M., Marszałek K., Eş I., Munekata P.E.S., Ferreira I., Barba F.J. (2019). Understanding the potential benefits of thyme and its derived products for food industry and consumer health: From extraction of value-added compounds to the evaluation of bioaccessibility, bioavailability, anti-inflammatory, and antimicrobial activities. Crit. Rev. Food Sci. Nutr..

[5] IUCN (2020). The IUCN Red List of Threatened Species. Version 2020-3. https://www.iucnredlist.org/.

[6] Roxo M., Zuzarte M., Gonçalves M.J., Alves-Silva J.M., Cavaleiro C., Cruz M.T., Salgueiro L. (2020). Antifungal and anti-inflammatory potential of the endangered aromatic plant *Thymus albicans*. Sci. Rep..

[7] Martins-Gomes C., Taghouti M., Schäfer J., Bunzel M., Silva A.M., Nunes F.M. (2018). Chemical characterization and bioactive properties of decoctions and hydroethanolic extracts of *Thymus carnosus* Boiss. J. Funct. Foods.

[8] Martins-Gomes C., Souto E.B., Cosme F., Nunes F.M., Silva A.M. (2019). *Thymus carnosus* extracts induce anti-proliferative activity in Caco-2 cells through mechanisms that involve cell cycle arrest and apoptosis. J. Funct. Foods.

[9] Figueiredo A.C., Pedro L.G., Barroso J.G., Trindade H., Sanches J., Oliveira C., Correia M. (2013). *Thymus capitellatus* Hoffmanns. & Link. Hortofruticult. Floric. Agrotec..

[10] Machado M., Dinis A.M., Santos-Rosa M., Alves V., Salgueiro L., Cavaleiro C., Sousa M.C. (2014). Activity of *Thymus capitellatus* volatile extract, 1,8-cineole and borneol against Leishmania species. Vet. Parasitol..

[11] Salgueiro L.R., Pinto E., Gonçalves M.J., Costa I., Palmeira A., Cavaleiro C., Pina-Vaz C., Rodrigues A.G., Martinez-de-Oliveira J. (2006). Antifungal activity of the essential oil of *Thymus capitellatus* against Candida, Aspergillus and dermatophyte strains. Flavour Fragr. J..

[12] Figueiredo A.C., Barroso J.G., Pedro L.G., Pais M.S.S., Scheffer J.J.C. (1993). The essential oils of two endemic Portuguese thyme species: *Thymus capitellatus* Hoffmanns. & Link and *T. lotocephalus* G. López & R. Morales. Flavour Fragr. J..

[13] Adzet T., Vila R., Canigueral S. (1988). Chromatographic analysis of polyphenols of some Iberian *Thymus*. J. Ethnopharmacol..

[14] Hernández L.M., Tomás-Barberán F.A., Tomás-Lorente F. (1987). A chemotaxonomic study of free flavone aglycones from some Iberian *Thymus* species. Biochem. Syst. Ecol..

[15] Husain S.Z., Markham K.R. (1981). The glycoflavone vicenin-2 its distribution in related genera within the Labiatae. Phytochemistry.

[16] Tavares L., Fortalezas S., Tyagi M., Barata D., Serra A.T., Martins Duarte C.M., Duarte R.O., Feliciano R.P., Bronze M.R., Espírito-Santo M.D. (2012). Bioactive compounds from endemic plants of Southwest Portugal: Inhibition of acetylcholinesterase and radical scavenging activities. Pharm. Biol..

[17] Taghouti M., Martins-Gomes C., Schäfer J., Félix L.M., Santos J.A., Bunzel M., Nunes F.M., Silva A.M. (2018). *Thymus pulegioides* L. as a rich source of antioxidant, anti-proliferative and neuroprotective phenolic compounds. Food Funct..

[18] Taghouti M., Martins-Gomes C., Schäfer J., Santos J.A., Bunzel M., Nunes F.M., Silva A.M. (2020). Chemical Characterization and Bioactivity of Extracts from *Thymus mastichina*: A *Thymus* with a Distinct Salvianolic Acid Composition. Antioxidants.

[19] Taghouti M., Martins-Gomes C., Félix L.M., Schäfer J., Santos J.A., Bunzel M., Nunes F.M., Silva A.M. (2020). Polyphenol composition and biological activity of *Thymus citriodorus* and *Thymus vulgaris*: Comparison with endemic Iberian Thymus species. Food Chem..

[20] Lim J., Zhang X., Ferruzzi M.G., Hamaker B.R. (2019). Starch digested product analysis by HPAEC reveals structural specificity of flavonoids in the inhibition of mammalian α-amylase and α-glucosidases. Food Chem..

[21] Rover M.R., Brown R.C. (2013). Quantification of total phenols in bio-oil using the Folin–Ciocalteu method. J. Anal. Appl. c.

[22] Silva A.M., Félix L.M., Teixeira I., Martins-Gomes C., Schäfer J., Souto E.B., Santos D.J., Bunzel M., Nunes F.M. (2021). Orange thyme: Phytochemical profiling, in vitro bioactivities of extracts and potential health benefits. Food Chem. X.

[23] Pereira O.R., Peres A.M., Silva A.M.S., Domingues M.R.M., Cardoso S.M. (2013). Simultaneous characterization and quantification of phenolic compounds in *Thymus* x *citriodorus* using a validated HPLC–UV and ESI–MS combined method. Food Res. Int..

[24] Ziani B.E.C., Heleno S.A., Bachari K., Dias M.I., Alves M.J., Barros L., Ferreira I.C.F.R. (2019). Phenolic compounds characterization by LC-DAD-ESI/MSn and bioactive properties of Thymus algeriensis Boiss. & Reut. and Ephedra alata Decne. Food Res. Int..

[25] Francescato L.N., Debenedetti S.L., Schwanz T.G., Bassani V.L., Henriques A.T. (2013). Identification of phenolic compounds in *Equisetum giganteum* by LC–ESI-MS/MS and a new approach to total flavonoid quantification. Talanta.

[26] Chikara S., Nagaprashantha L.D., Singhal J., Horne D., Awasthi S., Singhal S.S. (2018). Oxidative stress and dietary phytochemicals: Role in cancer chemoprevention and treatment. Cancer Lett..

[27] Elfawy H.A., Das B. (2019). Crosstalk between mitochondrial dysfunction, oxidative stress, and age related neurodegenerative disease: Etiologies and therapeutic strategies. Life Sci..

[28] Su Y., Li L. (2020). Structural characterization and antioxidant activity of polysaccharide from four auriculariales. Carbohydr. Polym..

[29] Kim I.-S., Yang M.-R., Lee O.-H., Kang S.-N. (2011). Antioxidant Activities of Hot Water Extracts from Various Spices. Int. J. Mol. Sci..

[30] Ozen T., Demirtas I., Aksit H. (2011). Determination of antioxidant activities of various extracts and essential oil compositions of *Thymus praecox* subsp. skorpilii var. skorpilii. Food Chem..

[31] Ertas A., Boga M., Yilmaz M.A., Yesil Y., Tel G., Temel H., Hasimi N., Gazioglu I., Ozturk M., Ugurlu P. (2015). A detailed study on the chemical and biological profiles of essential oil and methanol extract of *Thymus nummularius* (Anzer tea): Rosmarinic acid. Ind. Crops Prod..

[32] Afonso A.F., Pereira O.R., Neto R.T., Silva A.M.S., Cardoso S.M. (2017). Health-Promoting Effects of *Thymus herba-barona*, *Thymus pseudolanuginosus*, and *Thymus caespititius* Decoctions. Int. J. Mol. Sci..

[33] Barros L., Heleno S.A., Carvalho A.M., Ferreira I.C.F.R. (2010). Lamiaceae often used in Portuguese folk medicine as a source of powerful antioxidants: Vitamins and phenolics. LWT-Food Sci. Technol..

[34] Jaouadi R., Silva A.M.S., Boussaid M., Yahia I.B.H., Cardoso S.M., Zaouali Y. (2019). Differentiation of Phenolic Composition Among Tunisian *Thymus algeriensis* Boiss. et Reut. (Lamiaceae) Populations: Correlation to Bioactive Activities. Antioxidants.

[35] Ali F., Rahul, Jyoti S., Naz F., Ashafaq M., Shahid M., Siddique Y.H. (2019). Therapeutic potential of luteolin in transgenic Drosophila model of Alzheimer’s disease. Neurosci. Lett..

[36] Loesche A., Köwitsch A., Lucas S.D., Al-Halabi Z., Sippl W., Al-Harrasi A., Csuk R. (2019). Ursolic and oleanolic acid derivatives with cholinesterase inhibiting potential. Bioorg. Chem..

[37] Orhan I., Şenol F.S., Gülpinar A.R., Kartal M., Şekeroglu N., Deveci M., Kan Y., Şener B. (2009). Acetylcholinesterase inhibitory and antioxidant properties of *Cyclotrichium niveum*, *Thymus praecox* subsp. caucasicus var. caucasicus, *Echinacea purpurea* and *E. pallida*. Food Chem. Toxicol..

[38] Gülçin İ., Scozzafava A., Supuran C.T., Koksal Z., Turkan F., Çetinkaya S., Bingöl Z., Huyut Z., Alwasel S.H. (2016). Rosmarinic acid inhibits some metabolic enzymes including glutathione S-transferase, lactoperoxidase, acetylcholinesterase, butyrylcholinesterase and carbonic anhydrase isoenzymes. J. Enzym. Inhib. Med. Chem..

[39] Batiha G.E., Beshbishy A.M., Ikram M., Mulla Z.S., El-Hack M.E.A., Taha A.E., Algammal A.M., Elewa Y.H. (2020). The Pharmacological Activity, Biochemical Properties, and Pharmacokinetics of the Major Natural Polyphenolic Flavonoid: Quercetin. Foods.

[40] Taslimi P., Gulçin İ. (2017). Antidiabetic potential: In vitro inhibition effects of some natural phenolic compounds on α-glycosidase and α-amylase enzymes. J. Biochem. Mol. Toxicol..

[41] Angelis I.D., Turco L. (2011). Caco-2 Cells as a Model for Intestinal Absorption. Curr. Protoc. Toxicol..

[42] Riedl A., Schlederer M., Pudelko K., Stadler M., Walter S., Unterleuthner D., Unger C., Kramer N., Hengstschläger M., Kenner L. (2017). Comparison of cancer cells in 2D vs 3D culture reveals differences in AKT-mTOR-S6K signaling and drug responses. J. Cell Sci..

[43] Dehn P.F., White C.M., Conners D.E., Shipkey G., Cumbo T.A. (2004). Characterization of the human hepatocellular carcinoma (hepg2) cell line as an in vitro model for cadmium toxicity studies. Vitr. Cell. Dev. Biol. Anim..

[44] Lesjak M., Beara I., Simin N., Pintać D., Majkić T., Bekvalac K., Orčić D., Mimica-Dukić N. (2018). Antioxidant and anti-inflammatory activities of quercetin and its derivatives. J. Funct. Foods.

[45] Ramos A.A., Pereira-Wilson C., Collins A.R. (2010). Protective effects of Ursolic acid and Luteolin against oxidative DNA damage include enhancement of DNA repair in Caco-2 cells. Mutat. Res./Fundam. Mol. Mech. Mutagen..

[46] Song Y.S., Park C.M. (2014). Luteolin and luteolin-7-O-glucoside strengthen antioxidative potential through the modulation of Nrf2/MAPK mediated HO-1 signaling cascade in RAW 264.7 cells. Food Chem. Toxicol..

[47] Machado M., Felizardo C., Fernandes-Silva A.A., Nunes F.M., Barros A. (2013). Polyphenolic compounds, antioxidant activity and l-phenylalanine ammonia-lyase activity during ripening of olive cv. “Cobrançosa” under different irrigation regimes. Food Res. Int..

[48] Sreejayan, Rao M.N.A. (1997). Nitric oxide scavenging by curcuminoids. J. Pharm. Pharmacol..

[49] Tao H., Zhou J., Wu T., Cheng Z. (2014). High-Throughput Superoxide Anion Radical Scavenging Capacity Assay. J. Agric. Food Chem..

[50] Andreani T., Kiill C.P., de Souza A.L., Fangueiro J.F., Fernandes L., Doktorovova S., Santos D.L., Garcia M.L., Gremiao M.P., Souto E.B. (2014). Surface engineering of silica nanoparticles for oral insulin delivery: Characterization and cell toxicity studies. Colloids Surf. B Biointerfaces.

[51] Silva A.M., Martins-Gomes C., Coutinho T.E., Fangueiro J.F., Sanchez-Lopez E., Pashirova T.N., Andreani T., Souto E.B. (2019). Soft Cationic Nanoparticles for Drug Delivery: Production and Cytotoxicity of Solid Lipid Nanoparticles (SLNs). Appl. Sci..

